# Using synchrotron high-resolution powder X-ray diffraction for the structure determination of a new cocrystal formed by two active principle ingredients

**DOI:** 10.1107/S2053229624000639

**Published:** 2024-01-28

**Authors:** Mathieu Guerain, Natalia T. Correia, Luisa Roca-Paixão, Hubert Chevreau, Frederic Affouard

**Affiliations:** a Université de Lille, CNRS, INRAE, Centrale Lille, UMR 8207-UMET-Unité Matériaux et Transformations, F-59000 Lille, France; b Synchrotron SOLEIL, L Orme des Merisiers, Saint-Aubin, BP 48, 91192 Gif-sur-Yvette, France; J-PARC Center, Japan Atomic Energy Agency, Japan

**Keywords:** powder diffraction, API, carbamazepine, naproxen, cocrystal, PXRD, crystal structure, liquid-assisted grinding

## Abstract

The crystal structure of a new 1:1 cocrystal of carbamazepine and *S*-naproxen (C_15_H_12_N_2_O·C_14_H_14_O_3_) was solved from powder X-ray diffraction (PXRD). The PXRD pattern was measured at the high-resolution beamline CRISTAL at synchrotron SOLEIL (France). The structure was solved using Monte Carlo simulated annealing, then refined with Rietveld refinement. The positions of the H atoms were obtained from density functional theory (DFT) ground-state calculations. The symmetry is ortho­rhom­bic with the space group *P*2_1_2_1_2_1_ (No. 19) and the following lattice parameters: *a* = 33.5486 (9), *b* = 26.4223 (6), *c* = 5.3651 (10) Å and *V* = 4755.83 (19) Å^3^.

## Abstract 2

The crystal structure of a new 1:1 cocrystal of carbamazepine and *S*-naproxen was solved from powder X-ray diffraction. The powder pattern was measured at the high-resolution beamline CRISTAL at synchrotron SOLEIL (France). The positions of the H atoms were obtained from density functional theory (DFT) ground-state calculations.

## Introduction

In recent years, the design of functional pharmaceutical mol­ecular materials by the cocrystallization technique has attracted increasing inter­est (Friščić & Jones, 2010 ▸) when other classical approaches based, for example, on salt formation or metastable polymorphs are not possible. The strong development of this strategy has the consequence that many new active pharmaceutical ingredients (APIs) synthesized in the crystalline state exhibit poor solubility and bioavailability that is a major roadblock for pharmaceutical development. The aim is to construct an assembly of neutral multiple chemical species, in a stoichiometric ratio, in the same crystal lattice *via* weak supra­molecular inter­actions of various natures, such as van der Waals, hydrogen, halogen or π–π bonds. These multi­com­ponent materials in the crystalline solid state have an obvious inter­est in terms of stability, but also in improving many physicochemical properties of an API, such as its aqueous solubility, dissolution, hygroscopicity or bioavailability. Up to now, pharmaceutical cocrystals generally consist of an API and a coformer present in the same crystal lattice (Friščić & Jones, 2010 ▸; Vishweshwar *et al.*, 2006 ▸; Schultheiss & Newman, 2009 ▸; Brittain, 2013 ▸; Childs *et al.*, 2009 ▸), for example, paracetamol–piperazine (Oswald *et al.*, 2002 ▸), ibuprofen–nicotinamide (Berry *et al.*, 2008 ▸), carbamazepine–saccharin (Fleischman *et al.*, 2003 ▸), carbamazepine–tartaric acid (Guerain *et al.*, 2020 ▸), *etc*. In general, the coformer is not an API. In certain cases that are still quite rare, two APIs can be combined (Drozd *et al.*, 2017 ▸; Thakuria & Sarma, 2018 ▸), a situation which is of obvious inter­est for multi-therapy approaches.

Carbamazepine (CBZ, C_15_H_12_N_2_O, see Scheme 1), an API used as an anti-epileptic and analgesic drug, is a very common model system for the study of crystallization and cocrystallization (Childs *et al.*, 2009 ▸). CBZ is characterized by a rich polymorphism and five anhydrous crystalline forms (Grzesiak *et al.*, 2003 ▸; Rustichelli *et al.*, 2000 ▸; Arlin *et al.*, 2011 ▸) have been reported in the literature. The structure of the stable phase at ambient temperature and atmospheric pressure, named Form III [CBZ(III)], is the commercial form. It is monoclinic with the space group *P*2_1_/*n* and the following lattice parameters (Eccles *et al.*, 2011 ▸): *a* = 7.55, *b* = 11.186, *c* = 13.954 Å and β = 92.938°. 
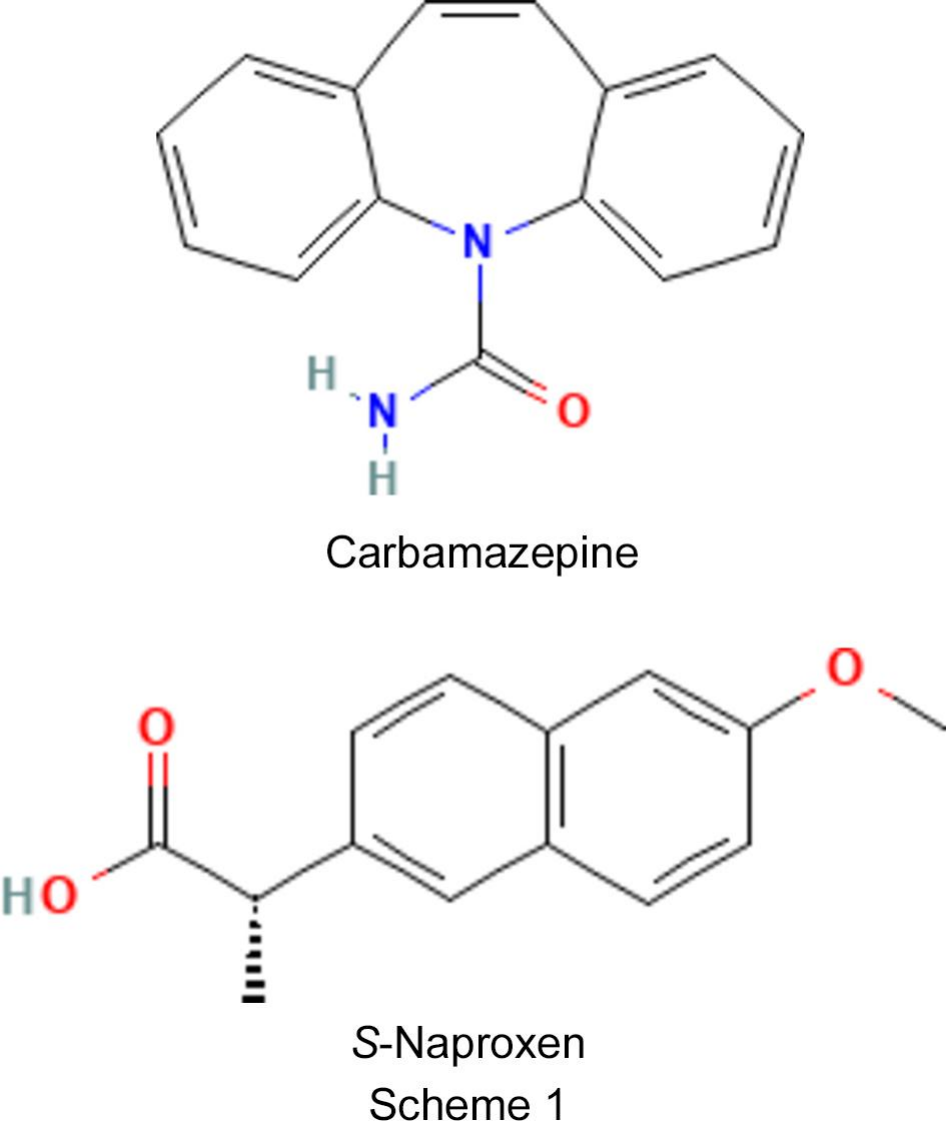


Due to its high polymorphism, and the hydrogen-bonding group offering the possibility of dimer formation, CBZ is also an excellent candidate for cocrystallization, as well as hydrate or solvate formation, as shown by examples in the literature (Vishweshwar *et al.*, 2006 ▸; Schultheiss & Newman, 2009 ▸; Childs *et al.*, 2009 ▸; Guerain *et al.*, 2020 ▸; Roca-Paixão *et al.*, 2019 ▸; Surov, Ramaza­nova *et al.*, 2023 ▸; Surov, Drozd *et al.*, 2023 ▸).

Naproxen (NAP, C_14_H_14_O_3_, see Scheme 1) is a nonsteroidal anti-inflammatory drug (NSAID) used to treat pain, men­strual cramps, inflammatory diseases, such as rheumatoid arthritis, gout and fever. The commercial form of *S*-naproxen (*S*-NAP) is the only known crystallographic form in the literature. It is monoclinic with the space group *P*2_1_ and the following lattice parameters (Tang *et al.*, 2015 ▸): *a* = 7.876, *b* = 5.783, *c* = 13.323 Å and β = 93.88°. *S*-NAP can form cocrystals with nicotinamide (Ando *et al.*, 2012 ▸; Neurohr *et al.*, 2015 ▸), isonicotinamide (Castro *et al.*, 2011 ▸) and proline (Tilborg *et al.*, 2013 ▸; Tumanova *et al.*, 2018 ▸), such mol­ecules being generally considered as safe.

In the present study it is shown that a cocrystal of CBZ and *S*-NAP (CBZ:*S*-NAP) can be been obtained from liquid-assisted grinding. It has been verified by coupling the search/match functionalities of *Highscore* software (Degen *et al.*, 2014 ▸) with the Cambridge Structural Database (CSD; Groom *et al.*, 2016 ▸), the Crystallographic Open Database (COD) (Gražulis *et al.*, 2009 ▸) and the PDF-2 database of the Inter­national Center for Diffraction Data (ICDD) (Gates-Rector & Blanton, 2019 ▸), that this cocrystal has not been referenced in the literature.

A pharmaceutical com­position that combines naproxen and carbamazepine is referred to in patent EA200200910 (A1) (Coe *et al.*, 2003 ▸; *A pharmaceutical com­position for treatment of acute, chronic pain and/or neuropathic pain and migraines*), but no reference is made to the elaboration of a cocrystal.

The present article aims to resolve the structure of the cocrystal CBZ:*S*-NAP obtained by liquid-assisted grinding in a 1:1 molar ratio. The structure was solved *ab initio* from powder X-ray diffraction using a direct-space approach (simulated annealing) and refined by the Rietveld method. The positions of the H atoms were estimated from energy minimization simulation.

## Experimental

### Cocrystal synthesis

*S*-Naproxen (purity higher than 98%) was purchased from Sigma–Aldrich and the material was used without any purification. The analysis of the powder X-ray diffraction pattern has shown that the commercial material is in the stable mono­clinic phase (CSD refcode COYRUD13; Tang *et al.*, 2015 ▸).

Carbamazepine (purity 99.8%) was purchased from Duchefa Farma BV and the material was used without any purification. The analysis of the powder X-ray diffraction pattern has shown that the commercial material is in the stable monoclinic phase (CSD refcode CBMZPN14; Eccles *et al.*, 2011 ▸).

The cocrystal was obtained by liquid-assisted grinding of 200 mg of a mixture of CBZ and *S*-NAP, in a 1:1 molar ratio, at 30 Hz for a period of 30 min, adding 20 µl of methanol to the mixture (Roca-Paixão *et al.*, 2019 ▸). Differential scanning calorimetry (DSC, Q1000, TA Instruments) reveals a single sharp endotherm associated with the melting of the synthesized pure cocrystal at *T*_m,onset_ = 125 °C, which demonstrates a decrease of the melting temperature com­pared to both parent com­pounds {*T*_m_(NAP) = 155 °C and *T*_m_[CBZ(III)] = 176 °C}. As is often the case with cocrystals obtained by grinding, and already observed in the case of carbamazepine, it was not possible to obtain a single crystal which would have facilitated the crystal structure determination (Roca-Paixão *et al.*, 2023 ▸; Guerain *et al.*, 2020 ▸).

### Data collection

The powder X-ray diffraction patterns were measured at the high-resolution powder diffraction beamline CRISTAL at the Synchrotron SOLEIL in France. The beamline is equipped with a 1D detector ‘MYTHEN2 X’. The selected energy was 18.4 keV, corresponding to a wavelength λ = 0.67132 Å, and a NIST standard LaB_6_ 660a sample was used for calibration. The cocrystal powder was enclosed in a borosilicate capillary (diameter 0.5 mm) and mounted on the goniometer head. The capillary was rotated during the experiments to reduce the effect of a possible preferential orientation. Data were collected at room temperature in the 1.5–50° 2θ range in less than 2 min to avoid radiation damage to the sample.

### Structure solution and refinement

Regarding the indexation, the profiles of 20 reflections with a 2θ angle lower than 15° were refined individually with the program *DASH* (David *et al.*, 2006 ▸) in order to obtain their 2θ angular positions. The 2θ values of these reflections were com­puted in the program *DICVOL* (Boultif & Louër, 2004 ▸) and a unique ortho­rhom­bic cell was obtained: *a* = 33.564 ± 0.001, *b* = 26.444 ± 0.001, *c* = 5.3666 ± 0.001 Å and *V* = 4763.22 ± 0.2 Å^3^. The calculated figures of merit are *M*(20) = 19.2 and *F*(20) = 136.3 (de Wolff *et al.*, 1968 ▸; Smith & Snyder, 1979 ▸).

Regarding the space-group determination, the *DASH* probabilistic approach (Markvardsen *et al.*, 2008 ▸), based on the systematic absences of Bragg peaks, was used. Eight individual peaks distributed over the whole 2θ range of the pattern were fitted to determine the peak-shape parameters, then the background, unit-cell and zero-point parameters were refined, and the most probable space group was calculated using Pawley refinement (Pawley, 1981 ▸). This method was repeated over ten times, each time on different sets of peaks. It led systematically to the space group *P*2_1_2_1_2_1_, which is the most probable space group for an ortho­rhom­bic cell, according to the CSD.

Using this space group (*P*2_1_2_1_2_1_) and the unit-cell parameters obtained previously, the X-ray diffraction pattern was refined using Pawley fitting (Pawley, 1981 ▸) with the program *DASH* (David *et al.*, 2006 ▸). The refinement was performed from 2θ = 1.5 to 15°. Again, eight individual peaks distributed over the whole 2θ range of the pattern were fitted to determine the peak-shape parameters. A five-term polynomial representing the background, the reflection intensities, the unit-cell parameters, the zero-point and the peak shape were refined. This led to a good correlation between the experimental diagram and the Pawley fitting to the profile, with χ^2^ = 23.34. This result was used for the structure solution. In order to determine a hypothetical structural model, the simulated annealing algorithm of the program *DASH* was used (David *et al.*, 2006 ▸).

Here, the *S*-NAP mol­ecule and the CBZ mol­ecule were retrieved from the CSD, *i.e.* from the monoclinic *S*-NAP phase model (Tang *et al.*, 2015 ▸) and from the monoclinic CBZ phase model (Eccles *et al.*, 2011 ▸), respectively. The volume calculated from the indexation (*V* = 4763.22 Å^3^) suggested the introduction of two mol­ecules of CBZ and two mol­ecules of *S*-NAP. The mol­ecules were introduced randomly in the cell. The restraints options used for the calculations did not modify the bond lengths and angles. The translation and orientation parameters of the mol­ecule in the cell, as well as the torsion angles, were defined as variables in the calculation. The maximum number of simulated annealing moves per run was fixed at 10 000 000 and led to the solution with a profile χ^2^ factor close to 56.2. As a result, this structure was used for Rietveld refinement.

From this structural model, rigid-body Rietveld refinement was performed using *DASH*. The refinement was performed in three steps: first, the global isotropic temperature factor, second, the translation and orientation parameters of the mol­ecule, and third, the five torsion angles. Strong restraints on the bond lengths and angles were applied. In particular, the naphthalene ring of the *S*-NAP mol­ecules and the benzene rings of the CBZ mol­ecules were kept planar. The lattice parameters and the background parameters were set free.

The structural model obtained from the simulated annealing was also minimized using periodic density functional theory with fixed-cell dispersion-corrected density func­tion­al theory (DFT-D) (Giannozzi *et al.*, 2009 ▸, 2017 ▸). In this minimization, the positions of the atoms were not con­strained. The Perdew–Burke–Ernzerhof (PBE) function was used with projector-augmented wave pseudopotentials and the Grimme D3 correction, as implemented in the pw.x exe­cutable of the *Quantum Espresso* program (Giannozzi *et al.*, 2009 ▸, 2017 ▸). Overall, only tiny differences are found between the atomic positions determined from the DFT minimization and from the Rietveld method (see supporting information).

**Figure 1:**
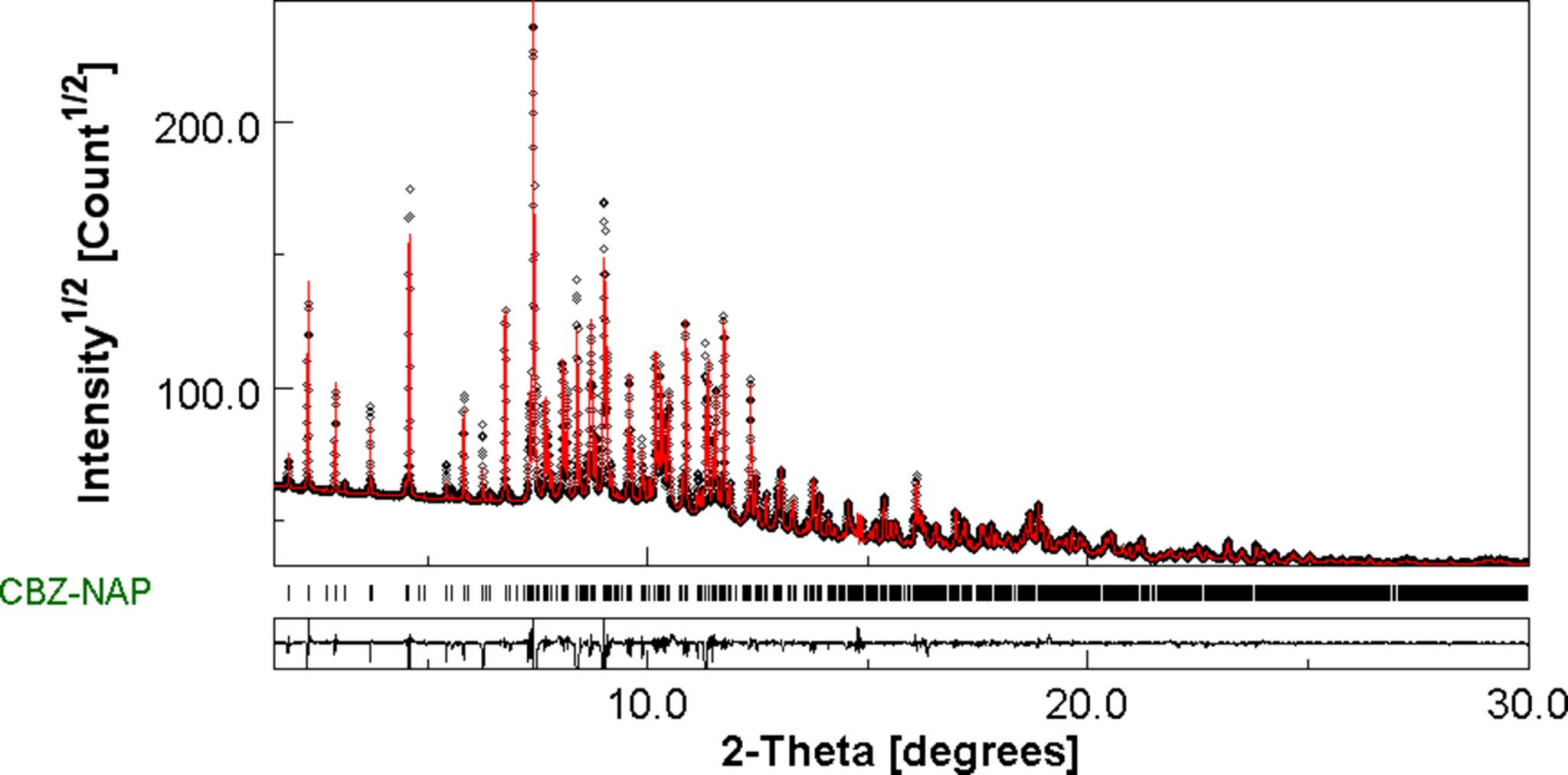
Final Rietveld plot of the CBZ:*S*-NAP cocrystal at room temperature. Observed intensities are indicated by dots, and solid lines represent the best-fit profile (upper trace) and the difference pattern (lower trace). The vertical bars correspond to the positions of the Bragg peaks.

Atomic coordinates found at the end of the Rietveld refinement were introduced in the programs *JANA2020* (Petrícek *et al.*, 2014 ▸) and *MAUD* (Materials Analysis Using Diffraction; Lutterotti, 2010 ▸). *JANA2020* was used to generate the more accurate and com­plete CIF possible and *MAUD* was used to graphically com­pare the calculated and experimental X-ray diffraction diagram. One can see in Fig. 1 ▸ the very reasonable agreement found between the calculated and the experimental X-ray diffraction diagram, reinforcing the validity of the reported structure.

At the end of the Rietveld refinements, the lattice parameters were *a* = 33.5486 (9), *b* = 26.4223 (6), *c* = 5.36515 (10) Å and *V* = 4755.83 (19) Å^3^. The final conventional Rietveld factors were *R* = 0.0413, *R*_wp_ = 0.0587, and *R*_exp _ = 0.0168. Such factors reflect the good correlation between the observed and simulated X-ray diffraction diagram, as shown in Fig. 1 ▸. Crystallographic data, profile and structural parameters are given in Table 1 ▸.

## Discussion

The structure obtained for the title cocrystal has a large unit-cell volume (4755.83 Å^3^), *i.e.* more than four times the unit-cell volume of commercial CBZ, and almost eight times the unit-cell volume of commercial *S*-NAP. This is related to the large lattice parameters *a* and *b*, of 33.5486 (9) and 26.4223 (6) Å, respectively.

Such lattice parameters are not surprising and are often obtained in the case of pharmaceutical cocrystals, such as ibuprofen–nicotinamide (Berry *et al.*, 2008 ▸), carbamazepine–indomethacine (Al Rahal *et al.*, 2020 ▸), carbamazepine–tartaric acid (Guerain *et al.*, 2020 ▸), naproxen–nicotinamide (Ando *et al.*, 2012 ▸; Neurohr *et al.*, 2015 ▸) and naproxen–isonicotinamide (Castro *et al.*, 2011 ▸).

**Figure 2:**
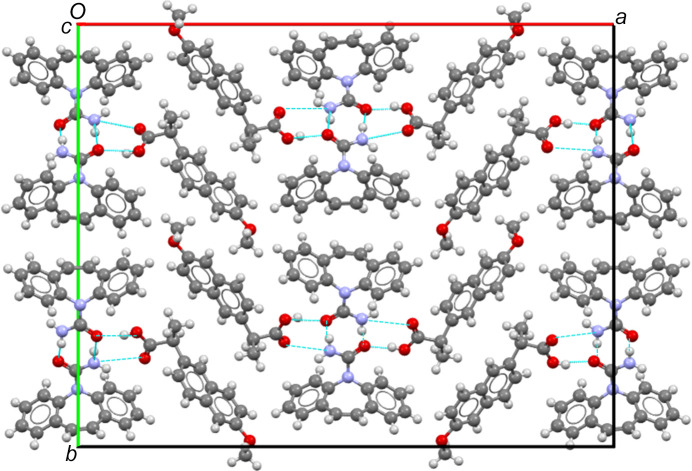
Visualization of the hydrogen-bond network of the CBZ:*S*-NAP cocrystal and projection of the unit cell along the [001] direction.

It is closely related to the intricate arrangement of CBZ and *S*-NAP mol­ecules within the crystal lattice (Fig. 2). The CBZ and *S*-NAP mol­ecules are stacked without orientation change along the *c* direction, leading to a rather small *c* parameter of 5.36515 (10) Å. However, the mol­ecular arrangement exhibits a greater com­plexity in the *a* and *b* directions. Along the *a* direction, an alternation pattern of CBZ and *S*-NAP mol­ecules is observed, with a 180° rotation between two *S*-NAP mol­ecules and a 180° rotation of the CBZ amine group between two CBZ mol­ecules, *i.e.* an alternation of a total of four mol­ecules (two CBZ and two *S*-NAP), contributing to a substantial large *a* unit-cell parameter [33.5486 (9) Å]. In the *b* direction, an alternation pattern of four mol­ecules of either CBZ or *S*-NAP is observed. This alternation is attributed to the dimer formation between CBZ mol­ecules, with each dimer experiencing a 180° rotation of the amine group. Such a dimer formation is commonly observed in cocrystals involving CBZ mol­ecules (Roca-Paixão *et al.*, 2023 ▸; Walsh *et al.*, 2003 ▸; Al Rahal *et al.*, 2020 ▸; Habgood *et al.*, 2010 ▸; Oliveira *et al.*, 2011 ▸). As regards the *S*-NAP mol­ecules, an inversion of the mol­ecules two by two is observed, with, between two inversions, a 180° rotation of the carb­oxy­lic acid group. These two different conformations of the *S*-NAP mol­ecules (by rotations of the groups along the C16—C17 and C30—C31 bonds) and the CBZ molecules (by rotations of the groups along the N1—C11 and N3—C54 bonds) are closely related to the com­plexity of the unit cell (see supporting information). As a consequence of these rotations, two symmetry-independent mol­ecules (ener­getically different) can be dis­tin­guished, leading to a crystal symmetry lower than for a theoretical structure without those rotations. Such rotations facilitate the dimer formation (no steric hindrance) and break the symmetry, so the mol­ecules forming the dimer are not identical.

This structural arrangement is linked to the hydrogen-bonding network of the cocrystal (Fig. 2 ▸). Notably, it is worth emphasizing that a single CBZ mol­ecule inter­acts through three different hydrogen bonds, involving the two H atoms of its amine group and its O atom:

(i) with another CBZ mol­ecule, with two N—H⋯O hydrogen bonds coming from one H atom of the amine group and forming a dimer as discussed previously;

(ii) with a *S*-NAP mol­ecule, with an N—H⋯O hydrogen bond between the second H atom of the amine group and the carb­oxy­lic acid group of *S*-NAP;

(iii) with the same *S*-NAP mol­ecule, with an O—H⋯O hydrogen bond between the O atoms of CBZ and the carb­oxy­lic acid group of *S*-NAP.

Consequently, the two *S*-NAP mol­ecules also form a dimer which is based on the dimer of two CBZ mol­ecules.

Also, the CBZ mol­ecules are bound by hydrogen bonds of type (i), while the inter­actions between the two mol­ecules com­posing the cocrystal are mainly related to hydrogen bonds of types (ii) and (iii), which bind the CBZ mol­ecule to the *S*-NAP mol­ecules. The whole forms a fairly rich and com­pact network of hydrogen bonds.

## Conclusion

In this work, the cocrystal CBZ:*S*-NAP was synthesized by the liquid-assisted grinding in a 1:1 molar ratio. A search in the PDF-2, CSD and COD databases shows that the crystallographic structure of this cocrystal was unknown in the literature. It was solved using powder diffraction experiments at the beamline CRISTAL at the Synchrotron SOLEIL in France. Indexation of the diagram, simulated annealing, theoretical calculations and Rietveld refinement led to an ortho­rhom­bic cocrystal with the space group *P*2_1_2_1_2_1_ (No. 19) and the following lattice parameters: *a* = 33.5486 (9), *b* = 26.4223 (6), *c* = 5.36515 (10) Å and *V* = 4755.83 (19) Å^3^.

## Supplementary Material

Crystal structure: contains datablock(s) global, I. DOI: 10.1107/S2053229624000639/oj3014sup1.cif

CIF from DFT calculations. DOI: 10.1107/S2053229624000639/oj3014sup2.txt

X-ray diagrams and vizualisation of the crystal structures. DOI: 10.1107/S2053229624000639/oj3014sup3.pdf

Supporting information file. DOI: 10.1107/S2053229624000639/oj3014Isup4.cml

CCDC reference: 2327306

## Figures and Tables

**Table 1:** Crystallographic data, profile and structural parameters for the CBZ:*S*-NAP cocrystal obtained after Rietveld refinement

Crystal data	
Chemical formula	C_15_H_12_N_2_O·C_14_H_14_O_3_
Mol­ecular weight (g mol^−1^)	933.1
Crystal system, space group	ortho­rhom­bic, *P*2_1_2_1_2_1_
Temperature (K)	293
*a*,*b*,*c* (Å)	33.5486 (9), 26.4223 (6), 5.36515 (10)
*V* (Å^3^)	4755.83 (19)
*Z*	4
*F*(000)	1968
μ (mm^−1^)	0.077
Specimen shape, size (mm)	Cylinder, 0.5
2θ range (°)	1.5–20
	
Data collection	
Beamline	CRISTAL (SOLEIL)
Specimen mounting	0.5 mm diameter Lindemann capillary
Data collection mode	Transmission
Scan method	Continuous scan
Radiation type	Synchrotron 18.47 KeV, λ = 0.67132 Å
Binning size (°2θ)	0.004
	
Refinement	
*R* factors	*R* = 0.0413, *R*_wp,nb_ = 0.0587, *R*_exp _ = 0.0168
