# Supplementary material for: Using synchrotron high-resolution powder X-ray diffraction for the structure determination of a new cocrystal formed by two active principle ingredients
Source: Acta Crystallogr C Struct Chem. 2024 Jan 28;80(Pt 2):37–42. doi: 10.1107/S2053229624000639 (PMC10844954; doi:10.1107/S2053229624000639)
Supplement: Supplementary file 3 [file c-80-00037-sup3.pdf]

STRUCTURAL CHEMISTRY

Volume 80 (2024)

Supporting information for article:

Using synchrotron high-resolution powder X-ray diffraction for the structure determination of a new cocrystal formed by two active principle ingredients

Mathieu Guerain, Natalia Correia, Luisa Roca-Paixão, Hubert Chevreau and Frederic Affouard

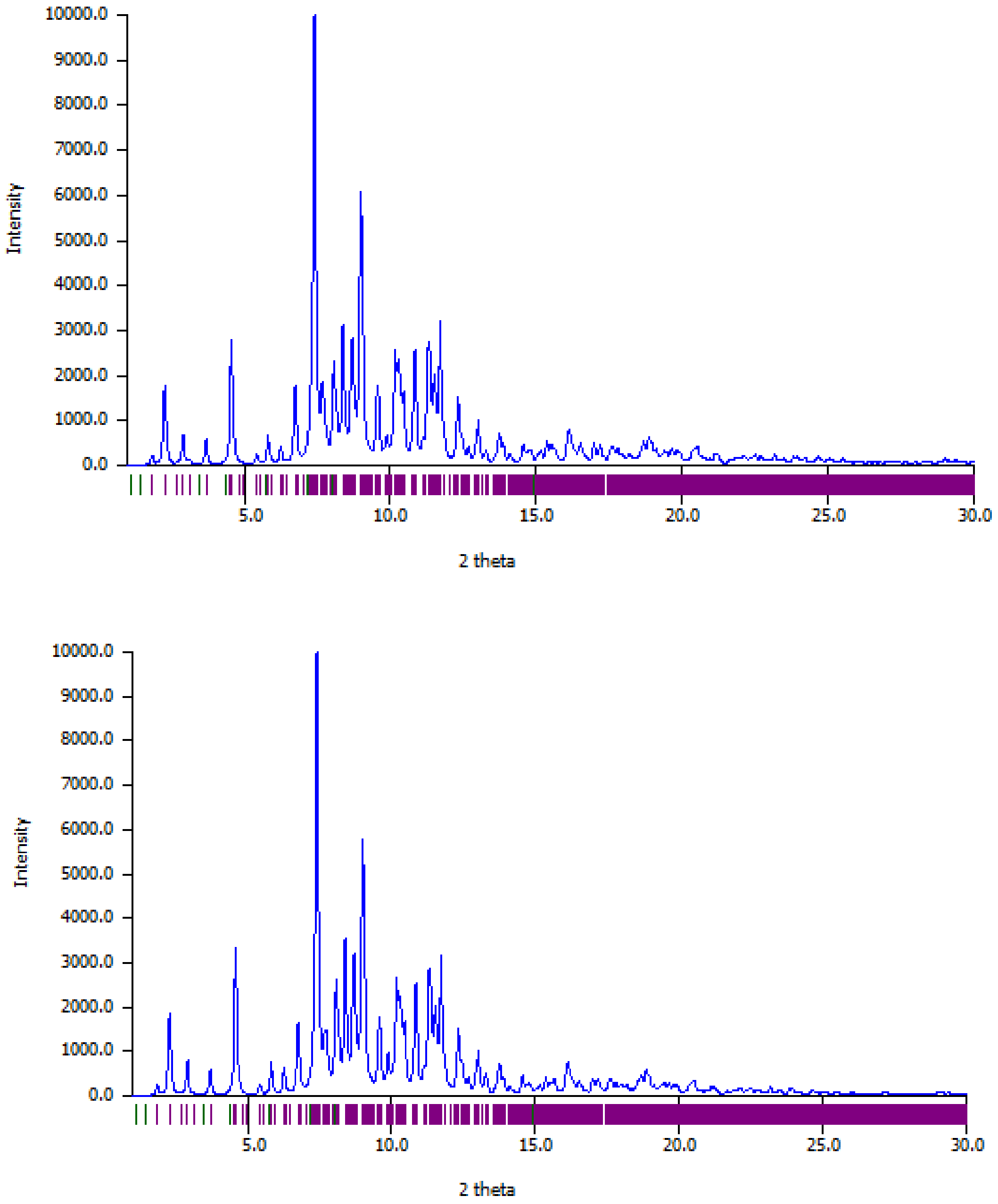

Figure S1 : X-Ray diagram obtained from the structural resolution of the co-crystal and Rietveld Refinement (up) and calculated X-Ray diagram obtained by DFT calculations (down)

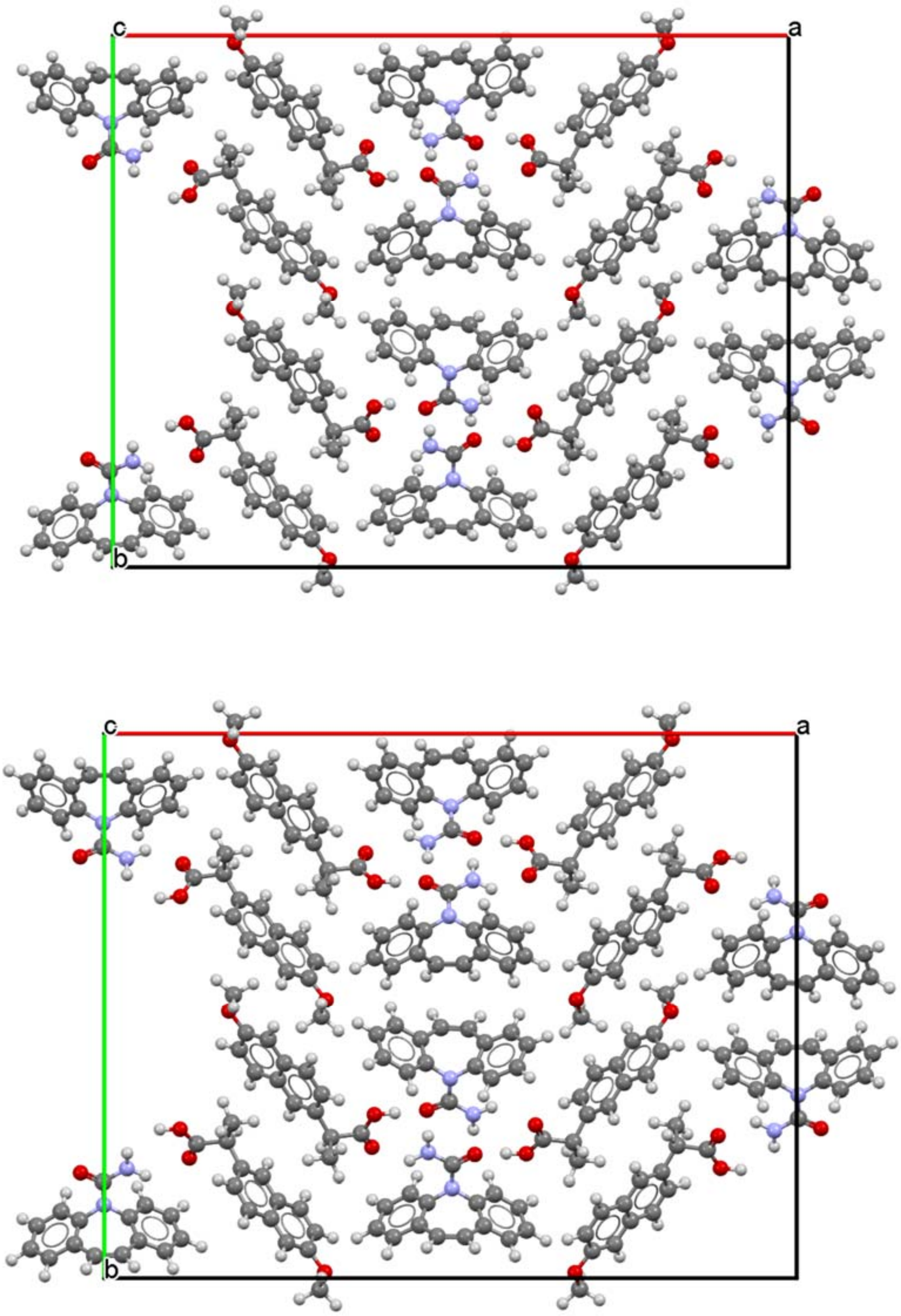

Figure S2 : Visualization of the cocrystal structure and projection of the unit cell along the [001] direction for the structure obtained after Rietveld Refinement Rietveld Refinement (up) and DFT calculation (down)

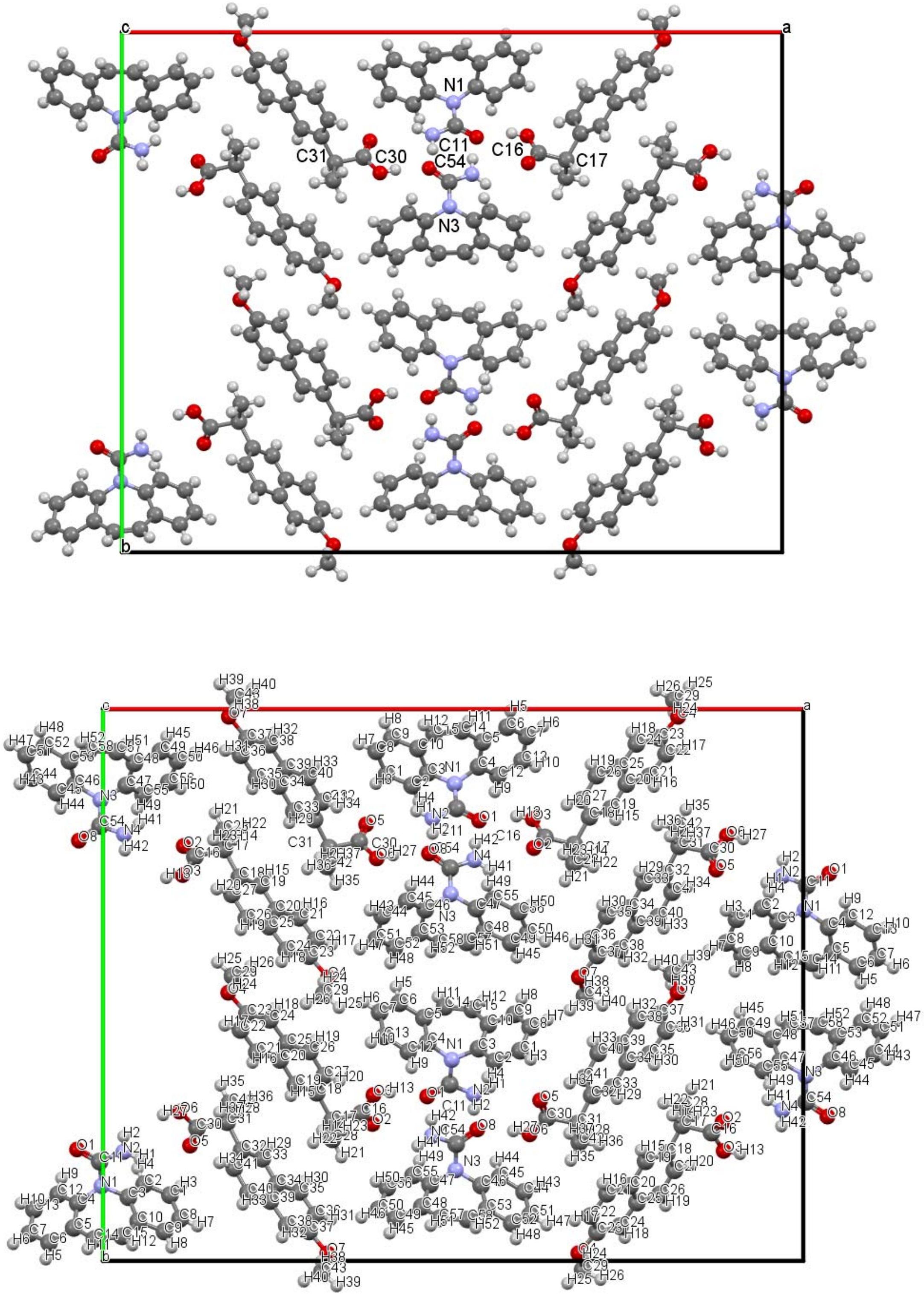

Figure S3 : Visualization of the cocrystal structure and projection of the unit cell along the [001]. Up : Only the C16—C17 and C30—C31 bonds are labelled. Down : All atoms are labelled.

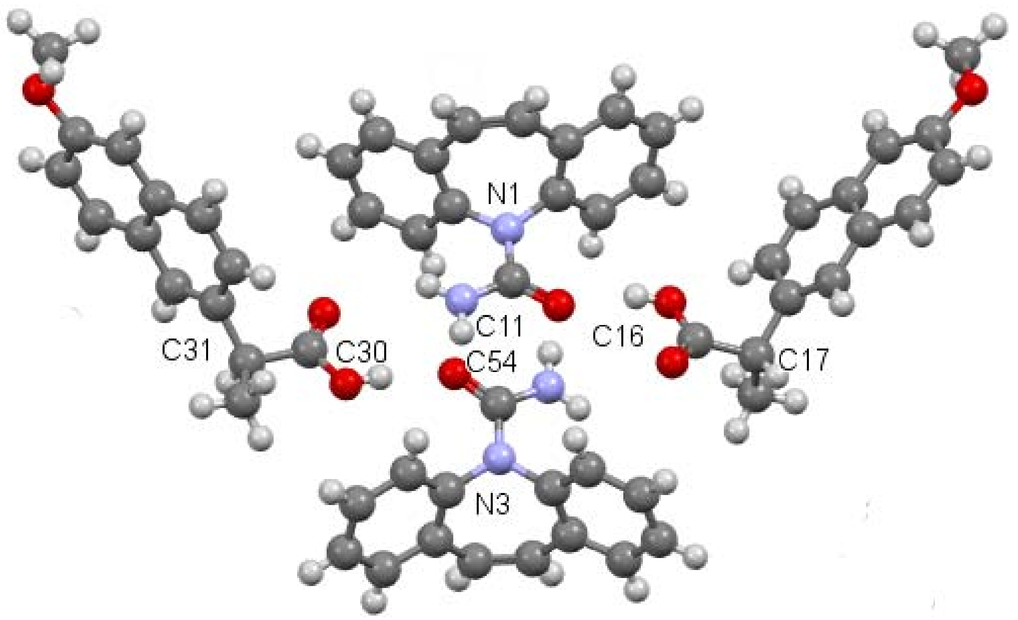

Figure S4 : Magnification of the CBZ and S-NAP molecules with the C16-C17 and C30-C31 bonds labelled.
